# MOG-expressing teratoma followed by MOG-IgG-positive optic neuritis

**DOI:** 10.1007/s00401-020-02236-5

**Published:** 2020-10-19

**Authors:** Brigitte Wildemann, Sven Jarius, Jonas Franz, Klemens Ruprecht, Markus Reindl, Christine Stadelmann

**Affiliations:** 1 Molecular Neuroimmunology Group, Department of Neurology, University of Heidelberg, Im Neuenheimer Feld 400, 69120 Heidelberg, Germany; 2 Institute of Neuropathology, University Medical Center Göttingen, Robert-Koch-Strasse 40, 37075 Göttingen, Germany; 3 Department of Neurology, Charité-Universitätsmedizin Berlin, Corporate Member of Freie Universität Berlin, Humboldt-Universität zu Berlin, and Berlin Institute of Health, Charitéplatz 1, 10117 Berlin, Germany; 4 Clinical Department of Neurology, Medical University of Innsbruck, Anichstrasse 35, 6020 Innsbruck, Austria

**Keywords:** Myelin oligodendrocyte glycoprotein (MOG), Antibodies, Optic neuritis, Ovarian teratoma

A paraneoplastic etiology has been reported in few patients with aquaporin-4 (AQP4)-IgG-seropositive neuromyelitis optica spectrum disorders (NMOSD), with lung and breast cancer being the most frequent associated malignancies [12]. Whether MOG encephalomyelitis (MOG-EM; also termed MOG antibody-associated disease [MOGAD]), a rare autoimmune disease characterized by serum immunoglobulin G antibodies (IgG) against myelin oligodendrocyte glycoprotein (MOG) and overlapping clinical and radiological features with both NMOSD and multiple sclerosis (MS) [4–7, 11], may occur in a paraneoplastic context is less well known. We report on a patient with oligosymptomatic MOG-EM emerging several months after detection and resection of an ovarian teratoma. Histopathology revealed neural tissue expressing MOG protein and accompanying immune cell infiltration within the teratoma, suggesting a possible paraneoplastic origin of MOG-EM in this case.

A 26-year-old woman with a history of Hashimoto thyroiditis developed right abdominal pain persisting for several weeks. A pelvic MRI showed a mass of 6 cm diameter adjacent to the right ovary displaying sebaceous components and calcifications. The right ovary was resected and histology confirmed a mature cystic teratoma. Eleven months later and within 3 weeks of an upper respiratory infection, the patient developed left-sided optic neuritis. Symptoms included mild eye pain, visual blurring, impaired color vision, along with a drop in visual acuity to 0.6. Cranial magnetic resonance imaging (MRI) revealed a normal optic nerve and a solitary small T2/fluid-attenuated inversion recovery lesion in the right frontal white matter; spinal MRI was unremarkable. CSF analysis disclosed normal cell and protein profiles and no oligoclonal bands. Serology for AQP4-IgG and NMDA-receptor-IgG was negative but positive for MOG-IgG (1:320). CSF was negative for MOG-IgG as typically the case in MOG-EM [7, 8]. Seropositivity was confirmed in three cell-based assays using either fixed or live cells expressing recombinant human full-length MOG protein as test substrate, two of which used Fc(γ)-specific secondary antibodies [3, 7, 10]. The symptoms resolved following high-dose intravenous methylprednisolone (IVMP) (5 × 1000 mg) and oral tapering over 4 weeks (initial 60 mg/d). Five months later, the patient noticed recurrence of pain upon eye movement and left-sided visual blurring compatible with a second episode of left-sided ON. Cranial MRI was unchanged and a repeat lumbar puncture disclosed no abnormalities except few activated lymphocytes. MOG-IgG was detectable at low titers (1:32; cut-off 1:10). Treatment with IVMP (3 × 1000 mg) led to recovery of visual acuity, while slight impairment of color vision persists to date. A further pelvic MRI performed within several months after the second ON did not show evidence of recurrent teratoma. Two and four months after the second attack and 18 and 20 months after removal of the teratoma, MOG-IgG had turned negative according to three assays. To investigate a possible paraneoplastic origin of MOG-EM in this patient, tissue blocks of the teratoma were examined neuropathologically.

Histological studies revealed a predominantly cystic, mature teratoma. In addition to well-differentiated epidermis and dermis with sebaceous glands, eccrine glands and hair follicles, the teratoma-contained neuroectodermal tissue with expression of glial fibrillary acidic protein (GFAP) (Fig. 1a–e). Moreover, mesodermal tissue, namely adipocytes and fibroblasts, was present. Significant entodermal tissue or undifferentiated components were not observed. A detailed examination of the neuroectodermal tissue showed oligodendrocytes and axons with intermittent myelination (Fig. 1f–p) as demonstrated by expression of oligodendroglial markers (CNPase, NogoA, BCAS1, NG2, p25, Olig2), among them markers of mature oligodendrocytes, such as myelin basic protein (MBP) and MOG. Also, scattered infiltration with CD4 + and CD8 + T-cells was observed. CD68 + as well as MHC II-expressing macrophages/activated microglia and CD1c + dendritic cells (DCs) were found in and immediately adjacent to the neuroectodermal tissue (Online Resources 1 and 2). Moreover, different DC populations were abundant in the lymphoid tissue (CD1c + , CD205 +) and epidermis (CD1a + , CD1c + , Langerin +) within the teratoma (Online Resource 2). Mutations/copy number variation in the MOG gene within the tumoral tissue could not be assessed in the available sample but should be evaluated in future studies of similar cases.

**Fig. 1:**
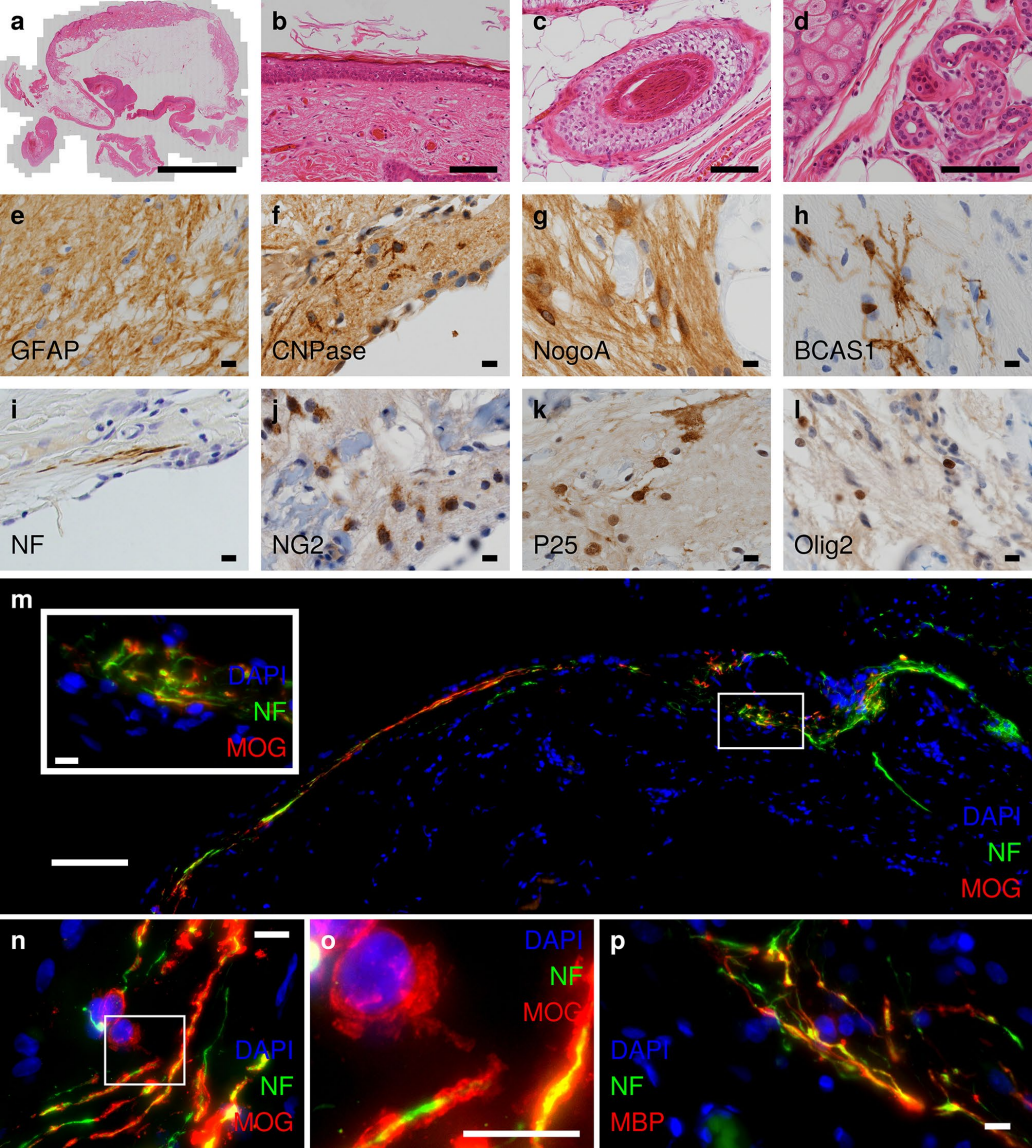
Mature ovarian teratoma (**a**) with typical ectodermal features such as epidermal differentiation (**b**), hair follicles (**c**) and eccrine and sebaceous glands (**d**) stained with hematoxylin and eosin. Parts of neuroectodermal tissue stained using GFAP antibodies (**e**). In these areas, oligodendrocytes were found expressing marker proteins such as CNPase (**f**), NogoA (**g**), BCAS-1 (**h**), NG2 (**j**), p25 (**k**) and Olig2 (**l**). They myelinated adjacent neurofilament (NF)-positive axons (**i**) with MOG (**m–o**) and also MBP-positive myelin sheaths (**p**). The axons showed long extensions detected by NF immunohistochemistry (**i**, **m–p**). Parts of the axons appear non-myelinated (**n**,** o**). Magnifications: 100 × (**a**); 400 × (**b-m,p**) and 1000 × (oil objective) (**n,** **o**). Scale bars: 10 mm (**a**); 100 µm (**b**, **c**, **d**, **m**); 10 µm **(e**–**l**, inset **m**,**n–p**). Z-depth: 8.74 µm (maximum intensity projection of 38 focal planes at a z-distance of 0.23 µm). Stainings were performed as previously described [2]

This patient developed two rather mild episodes of MOG-IgG-associated ON 11 and 16 months after diagnosis of an ovarian teratoma, which expressed MOG and other myelin proteins. Considering the latency of less than 1 year between removal of the tumor and ON, a humoral immune response directed against ectopic MOG expressed in the teratoma may have triggered remote neurologic autoimmunity in our patient. The infection preceding the first episode of ON might have facilitated the onset of MOG-EM. Infections, possibly by general immune cell activation or altering blood/brain barrier permeability, have been shown to frequently precede attacks in MOG-EM and AQP4-IgG-positive NMOSD [4, 6, 9]. A paraneoplastic etiology of MOG-EM in this case is further supported by the immune cell infiltration of the tumor and the fact that resection of the teratoma was followed by disappearance of MOG-IgG in the long-term course. An association of MOG-EM with ovarian teratoma has been previously observed by us and others in 2/77 patients from two cohorts [1, 4, 6] (Table 1). However, different from our patient, MOG expression within the teratoma was not studied in the previous cases. While no data are available in one case, MOG-IgG also disappeared after tumor removal in the other (testing performed by us after publication of [4] using two independent assays; sample taken 42 months after onset of MOG-EM and 40 months after teratoma resection; initial MOG-IgG titer was 1:10,240). Mature cystic teratomas have been reported to represent at least 10% of all ovarian tumors and to occur mostly in women of reproductive age. They often grow slowly at a mean rate of 1.8 mm/year and are often detected incidentally at routine pelvic ultrasound examination in asymptomatic women. Given the recent observations on a strong association of NMDAR-IgG-positive encephalitis with teratoma as well as reports on paraneoplastic NMOSD, studies systematically evaluating the prevalence of teratoma in MOG-IgG-positive adult women may be warranted. Our findings endorse the suggestion to test for MOG-IgG in patients with demyelinating disorders of the CNS and co-existing teratoma included in the recent international consensus recommendations on MOG-IgG testing [5].

**Table 1:** Clinical and serological findings in patients with MOG encephalomyelitis and teratoma

Case	Age at onset of MOG-EM/sex/origin	Leading symptom	Teratoma classification	MOG antigen present in tumor tissue	Relation of teratoma diagnosis and MOG-EM onset	Neurological outcome	MOG-IgG at last follow-up	NMDAR-antibodies in serum and CSF	Additional remarks
1 (present case)	27/f/Cauc	Recurrent ON	Benign, mature cystic ovarian teratoma	Yes	11 months prior to onset	Full recovery	Disappearance (initial titer 1:320)	Negative	
2 (Ref. 4, 6)	18/f/Cauc	LETM, BSTE	Benign, mature cystic ovarian teratoma	Not reported	2 months after onset	Almost full recovery	Disappearance (initial titer 1:10,240)	Negative	Diagnosis of retroperitoneal ganglioneuroma shortly after onset of MOG-EM
3 (Ref. 1)	16/f/Cauc	LETM, encephalopathy	Ovarian teratoma	Not reported	At onset?	Full recovery	Not reported (initial titer 1:640)	Negative	

*BSTE* brain stem encephalitis, *Cauc* caucasian, *CSF* cerebrospinal fluid, *EM* encephalomyelitis,  *f* female, *LETM* longitudinally extensive transverse myelitis, *MOG* myelin oligodendrocyte glycoprotein, *ON* optic neuritis

## Electronic supplementary material

Below is the link to the electronic supplementary material.

Supplementary file1 (PDF 7893 kb)

Supplementary file2 (PDF 19631 kb)
