# Supplementary material for: MOG-expressing teratoma followed by MOG-IgG-positive optic neuritis
Source: Acta Neuropathol. 2020 Oct 19;141(1):127–31. doi: 10.1007/s00401-020-02236-5 (PMC7785547; doi:10.1007/s00401-020-02236-5)
Supplement: Supplementary file 1 — Supplementary file1 (PDF 7893 kb) [file 401_2020_2236_MOESM1_ESM.pdf]

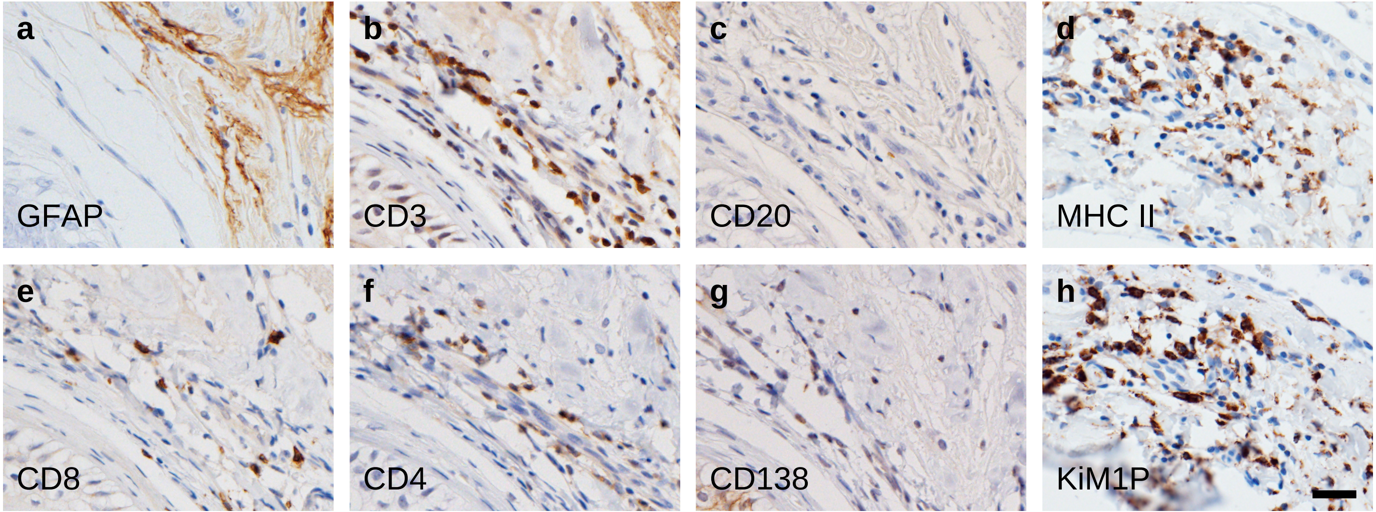
a
GFAP
b
CD3
c
CD20
d
MHC II
e
CD8
f
CD4
g
CD138
h
KiM1P

Online Resource 1. Inflammatory infiltrate directly associated with the CNS-like tissue component. CNS-like tissue identified by GFAP (**a**) was infiltrated by a mixed T-cell infiltrate (**b,e,f**) while CD20-positive B-cells (**c**) and CD138-positive plasma cells (**g**) were not observed in this area. Macrophages and DCs expressing MHC-II (**d**) and CD68 (clone KiM1P) (**h**) were frequently seen in the CNS-like tissue component. Scale bar length: 20 μm.
