# Supplementary material for: MOG-expressing teratoma followed by MOG-IgG-positive optic neuritis
Source: Acta Neuropathol. 2020 Oct 19;141(1):127–31. doi: 10.1007/s00401-020-02236-5 (PMC7785547; doi:10.1007/s00401-020-02236-5)
Supplement: Supplementary file 2 — Supplementary file2 (PDF 19631 kb) [file 401_2020_2236_MOESM2_ESM.pdf]

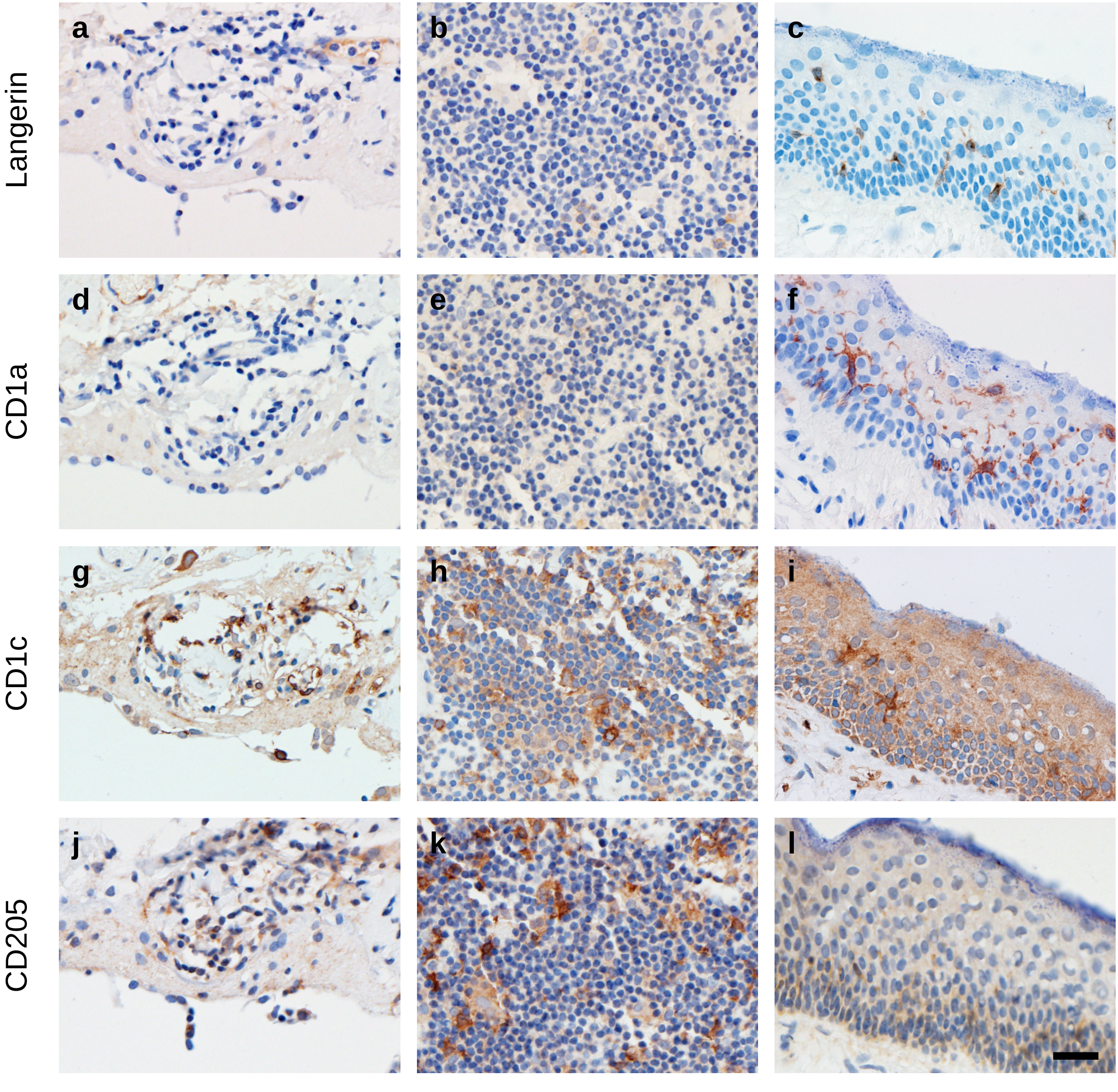
Langerin
CD1a
CD1c
CD205
a
b
c
d
e
f
g
h
i
j
k
l

Online Resource 2. Dendritic cells in the ovarian teratoma. Directly associated with the CNS-like tissue (**a,d,g,j**) a small perivascular inflammatory infiltrate did not harbor typical Langerin- or CD1a-positive dendritic cells (DCs), as regularly found in the epidermis. Also, no CD205-positive classical DCs were observed. However, a few CD1c/BDCA-1-positive DCs were identified. In teratoma-associated lymphoid tissue classical DC subsets expressing CD1c/BDCA-1 as well as classical DCs expressing CD205, but no CD1a- and Langerin-positive DCs, were found (**b,e,h,k**). In the epidermis of teratoma-derived skin tissue DCs expressing CD1a, CD1c and Langerin were present, being negative for CD205 (**c,f,i,l**). Scale bar length: 20 µm.
